# Comparison of Ti-Based Coatings on Silicon Nanowires for Phosphopeptide Enrichment and Their Laser Assisted Desorption/Ionization Mass Spectrometry Detection

**DOI:** 10.3390/nano7090272

**Published:** 2017-09-15

**Authors:** Ievgen Kurylo, Abderrahmane Hamdi, Ahmed Addad, Rabah Boukherroub, Yannick Coffinier

**Affiliations:** 1 University Lille, CNRS, Centrale Lille, ISEN, University Valenciennes, IEMN, UMR CNRS 8520, Avenue Poincaré, BP 60069, 59652 Villeneuve d’Ascq, France; ievgen.kurylo@gmail.com (I.K.); hamdiabderrahmen11@gmail.com (A.H.); rabah.boukherroub@univ-lille1.fr (R.B.); 2 Laboratory of Semi-Conductors, Nano-Structures and Advanced Technologies, Research and Technology Centre of Energy, Borj-Cedria Science and Technology Park, BP 95, 2050 Hammam-Lif, Tunisia; 3 Faculty of Science of Bizerte, University of Carthage, 7021 Zarzouna, Tunisia; 4 Unité Matériaux et Transformations (UMET), UMR CNRS 8207, Université Lille1, Cité Scientifique, 59655 Villeneuve d’Ascq, France; ahmed.addad@univ-lille1.fr

**Keywords:** silicon nanowires, titania, phosphopeptides, LDI-MS, serum

## Abstract

We created different TiO_2_-based coatings on silicon nanowires (SiNWs) by using either thermal metallization or atomic layer deposition (ALD). The fabricated surfaces were characterized by X-ray photoelectron spectroscopy (XPS), energy dispersive X-ray spectroscopy (EDX), and reflectivity measurements. Surfaces with different TiO_2_ based coating thicknesses were then used for phosphopeptide enrichment and subsequent detection by laser desorption/ionization mass spectrometry (LDI-MS). Results showed that the best enrichment and LDI-MS detection were obtained using the silicon nanowires covered with 10 nm of oxidized Ti deposited by means of thermal evaporation. This sample was also able to perform phosphopeptide enrichment and MS detection from serum.

## 1. Introduction

The protein phosphorylation event is one of the key regulators of intracellular biological processes and may be one of the most studied post-translational modifications (PTM) [1,2]. Indeed, the phosphorylation of amino-acid residues allows the regulation of several biological processes such as metabolism, transcriptional and translational regulation, and the degradation of proteins. It is also involved in the phenomenon of homeostasis, in cellular signaling and in cell communication, proliferation, differentiation and survival [3,4]. These transient and reversible PTMs provoke changes in the conformation of a protein, and so have a strong influence on its activity and interactions with the environment within a very short time frame. Because phosphorylation is a reversible phenomenon, protein activities can be finely controlled by either phosphorylation (using kinase) or by dephosphorylation (using phosphatase) in response to cellular or environmental stimuli. Most of the phosphorylation events are mainly realized with serine, threonine, or tyrosine amino-acids residues. However, despite their ubiquitous and crucial roles, phosphoproteins are generally present in low abundance. Indeed, the phosphorylated forms of individual proteins are present at much lower levels compared to their native counterparts. Within the phosphoproteome, most of the phosphorylated proteins are transiently phosphorylated following pivotal cellular signals, metabolic disorders or diseases, whereas some others are constitutively modified [5].

Since phosphorylation events help to maintain the living cell’s dynamics, the development of performant analytical methods for the detection/characterization of phosphorylated peptides is, obviously, crucial. Since phosphoproteins are present in relative low abundance with low phosphorylation stoichiometry, the need for sensitive and highly specific analytical strategies to characterize these PTMs is therefore crucial. In that context, phosphoproteins are mainly characterized, after proteolytic processing, through the use of mass spectrometry (MS).

However, phosphopeptide detection through MS is still challenging because of their lower ionization efficiency, which results in lower signal intensities, especially in the presence of non-phosphorylated peptide ions. Therefore, there is a clear need for efficient enrichment of the phosphorylated proteins or peptides, prior to MS analysis, in order to increase the sensitivity and obtain better phosphoproteome characterization. Many different phosphoproteomic enrichment strategies are currently used, and these depend heavily on the type of sample to be studied. Among them are: immunoprecipitation (immobilized anti-phosphoserine (pS), anti-phosphothreonine (pT) or anti-phosphotyrosine (pY) antibodies on magnetic beads) [6,7]; chemical derivatization (β-elimination, phosphoramidate-based chemistry) [8]; and IMAC (immobilized metallic ion affinity chromatography) [9,10] or MOAC (metal oxide affinity chromatography) based chromatographies [11,12,13]. IMAC adsorbents chelating Ga^3+^, Ti^4+^, Ni^2+^, or Zr^4+^ have been widely utilized for the enrichment of phosphopeptides in phosphoproteome analysis [14]. However, the main drawback of IMAC is the non-specific interaction of acidic amino acid and histidine residues [15]. MOAC is, therefore, preferred to IMAC, and metal oxides such as TiO_2_ and ZrO_2_ have been used for the enrichment of phosphopeptides due to the unique affinity between titania or zirconia and phosphopeptides [14].

However, in all these cases, phosphopeptide enrichment is mainly achieved through an “off-plate” sample preparation procedure (capture and elution steps) prior to MS analysis. Nevertheless, “on-plate” enrichment was developed to perform in situ phosphopeptide enrichment followed by rinsing steps and organic matrix deposition (DHB) prior to matrix-assisted laser desorption ionization mass spectrometry (MALDI-MS) detection [16]. This approach consists of modifying a MALDI target plate with spots of a material known to enrich phosphopeptides. In this case, the employed materials are similar to those used in IMAC e.g., polymer brushes made of poly(2-hydroxyethyl methacrylate) (pHEMA) derivatized with Fe(III)-nitriloacetate (NTA) or in MOAC e.g., a thin film of anatase titanium [16,17]. Enrichment is then achieved directly on the stainless steel MS target, and the non-specifically bounded peptides are removed using appropriate washing protocols.

In parallel, the development of matrix-free laser desorption/ionization mass spectrometry (LDI-MS) methods, based on micro- and nanostructured laser desorption ionization (LDI) targets, were proposed to eliminate the need for co-crystallization of the sample with an organic MALDI matrix. Among the main recognized advantages of a surface laser desorption/ionization (SALDI) matrix-free soft ionization method is the avoidance of intense matrix peaks present at low mass range of the spectra, increasing the background while lowering the sensitivity of detection of small molecules [18]. One of the most notable developments in this domain has been the development of desorption/ionization on porous silicon (DIOS), realized by the Siuzdak group, which uses an etched *p*-type Si target [19]. For certain classes of analytes at least, SALDI-MS equaled, or even exceeded, the sensitivity of classical MALDI-MS.

We have recently demonstrated that silicon nanowires (SiNWs) decorated with Cu/CuO particles can be used for “on-plate” His-tag peptide enrichment from serum spiked with His-tagged (*m*/*z* 1770) and untagged (*m*/*z* 905) peptides. In this case, Cu^2+^, contained in copper oxide, can interact with unprotonated (pH 7) nitrogen from imidazole groups (His*6-tag). The matrix-free LDI-MS detection was of a sensitivity down to 10 fmol/µL [20]. Concerning the phosphopeptides’ enrichment and their subsequent detection by SALDI-MS, TiO_2_ nanoparticles spots on an aluminum foil-based surface, thereby combining specific capture and mass spectrometry analysis, was used. The TiO_2_ was used as a specific affinity for in situ enrichment of phosphopeptides and for SALDI-MS analysis with lower sensitivity (few pmole) [21]. In the same vein, we proposed performing phosphorylated peptide enrichment and subsequent SALDI-MS detection. To do so, SiNWs made using metal-assisted chemical etching (MACE) were covered by TiO_2_ layers through thermal evaporation or atomic layer deposition techniques (ALD), respectively. Then, an equimolar mixture composed of non-phosphorylated peptides and mono- and diphosphorylated peptides was used to assess the performance of our interfaces as an efficient “on-plate” enrichment method and sensitive matrix-free LDI-MS technique.

## 2. Results and Discussion

**Figure 1:**
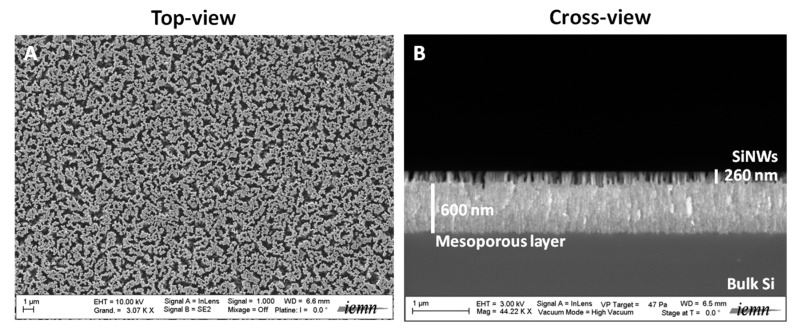
Top-view (**A**), (scale bar = 1 µm) and cross-view (**B**), (scale bar = 1 µm) SEM images of SiNWs.

The SiNWs investigated in this work were prepared using the MACE technique following a protocol already described in previous studies [22,23]. By adjusting reaction parameters such as time, temperature and reagent concentrations, various silicon surface morphologies can be achieved that present different nanostructure length, diameter, and overall porosity [22,24,25]. This method displays several advantages over others, as it takes place at room temperature and does not require any specific and expensive equipment. This straightforward preparation technique allows the generation of nanostructured surfaces presenting reproducible structural properties. MACE is also cheap and easy to undertake and scale up. The morphology of the SiNWs studied here consisted of a mesoporous film (600 nm thick) surmounted by porous silicon nanostructures (260 nm high), as seen in the SEM images in Figure 1 [22]. These SiNWs had already been used for the realization of LDI of peptides and various other small compounds and their subsequent mass spectrometry detection [22,23,26]. The LDI-MS mechanism involved on such silicon nanostructures is, in essence, based on the thermal mechanism [27]. Indeed, the silicon nanostructures (band gap: 1.1 eV) will absorb photons from the UV laser (λ = 355 nm, 3.68 eV). Then, the energy is transferred to the deposited analytes for desorption and ionization, allowing their MS detection.

Here, we introduced Ti^4+^ ions on SiNWs through the deposition of O_2_ films for specific enrichment of phosphopeptides. To do so, two strategies were used. The first was the use of metallization (by thermal evaporation) of titanium films (5 and 10 nm) with atmospheric oxidation to form a TiO_2_ overlayer (called oxidized Ti-SiNWs). The second was the direct deposition of three different thicknesses of TiO_2_ (2 nm, 5 nm and 10 nm) on silicon nanostructures by atomic layer deposition (ALD).

**Figure 2:**
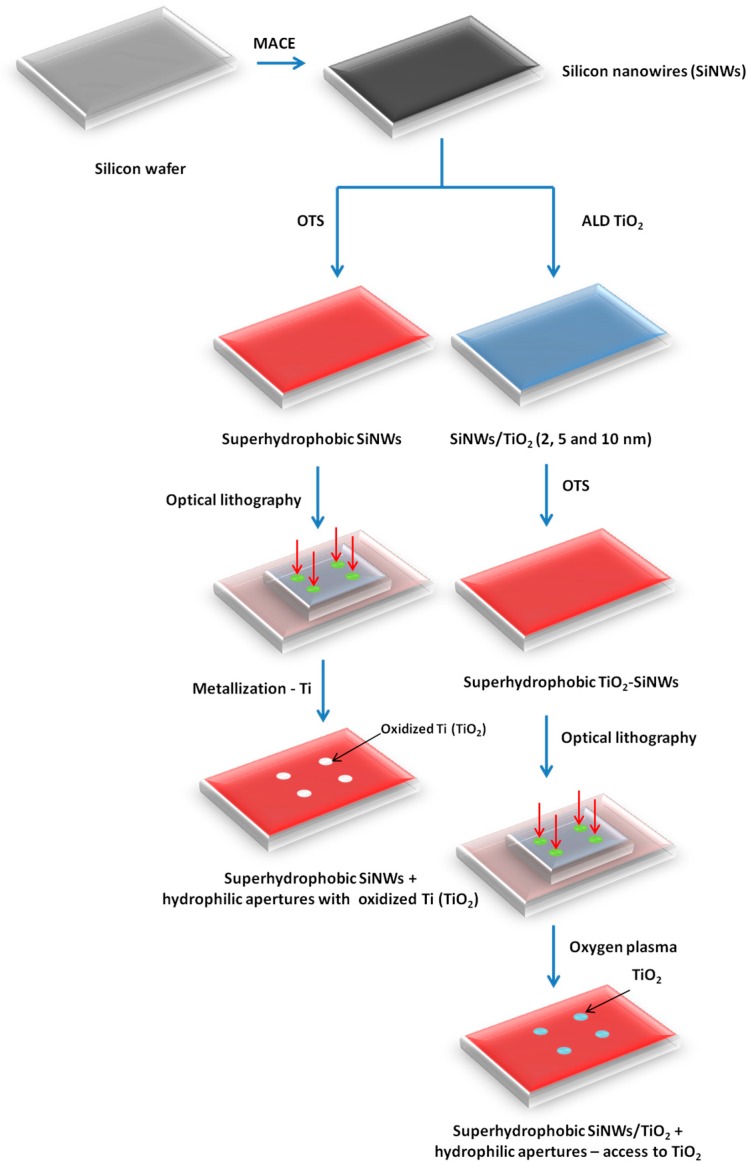
Schematic representation of the different steps to obtain TiO_2_-SiNW and oxidized Ti-SiNW surfaces.

Freshly prepared SiNWs and TiO_2_-SiNWs were chemically modified by silanization using octadecyltrichlorosilane (OTS). After the reaction, all SiNWs presented superhydrophobic behavior, as already shown in previous studies [24]. Then, photolithography was used to either metalize SiNWs on specific locations (oxidized Ti deposition) or to locally degrade the hydrophobic layer (OTS), thus creating specific hydrophilic apertures making TiO_2_ layers accessible to interaction with phosphate groups. Wetting contrast allowed liquid droplet confinement, increasing the sensitivity of detection. Figure 2 represents the two approaches used in this study to obtain accessible TiO_2_ coatings on SiNWs.

**Figure 3:**
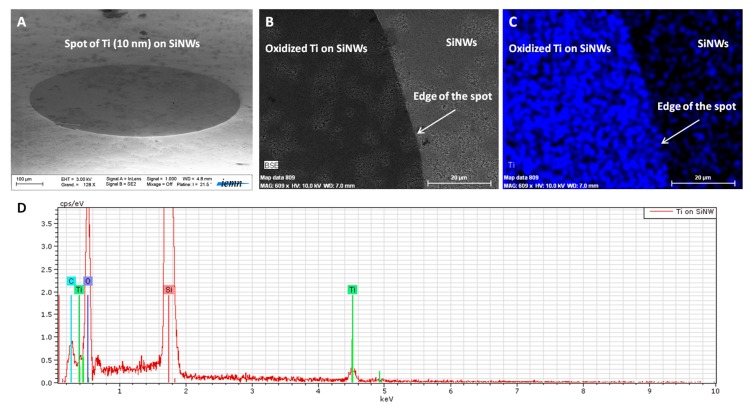
SEM image of oxidized Ti (10 nm) spot on SiNWs (**A**), a zoom showing the edge of the spot of Ti (**B**), its EDX mapping (**C**), with blue color corresponding to Ti element and the EDX spectrum of the spot (**D**).

To confirm the presence of TiO_2_ on both surfaces, we performed energy dispersive X-ray spectroscopy (EDX) analyses on the obtained interfaces. Figure 3 shows how EDX was performed on SiNWs presenting oxidized Ti spots (800 µm in diameter). On Figure 3A, we can clearly identify the oxidized Ti spot on the SiNWs’ surface. Figure 3B,C shows the SEM image of the edge of the oxidized Ti-SiNWs’ spot (dark area) and the corresponding EDX mapping, respectively. The blue color corresponds to the Ti element. The EDX spectrum, displayed in Figure 3D, confirmed the presence of the Ti, O, and Si elements.

**Figure 4:**
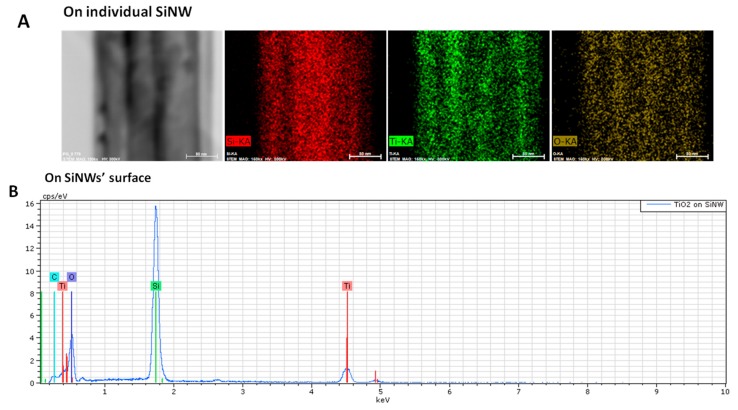
EDX mapping and analysis of TiO_2 (10 nm)_-SiNW surfaces on individual TiO_2_-SiNW (**A**) and on the TiO_2_-SiNWs’ surface (**B**). The scale bars in (**A**) correspond to 80 nm.

EDX analysis was also performed on TiO_2_-SiNWs. On Figure 4A, we can clearly see that an individual SiNW is uniformly covered by a TiO_2_ layer, proving the conformal deposition of TiO_2_ on SiNW obtained by ALD. EDX analysis on a larger scale showed the whole coverage of SiNWs by the TiO_2_ layer (Figure 4B). EDX mapping and analysis confirmed the presence of Ti and TiO_2_ around the SiNWs.

**Figure 5:**
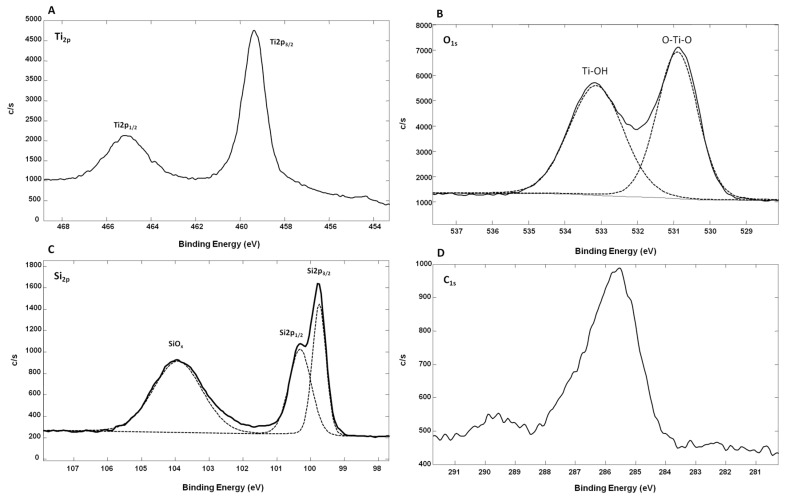
High-resolution XPS spectra of the oxidized T-SiNWs’ (10 nm) surface, with Ti_2p_ (**A**), O_1s_ (**B**), Si_2p_ (**C**) and C_1s_ (**D**). (c/s = counts/second).

In addition, and to better assess the chemical state of Ti, X-ray photoelectron spectroscopy (XPS) analyses were also conducted. Figure 5 shows the XPS spectra from oxidized Ti-SiNWs. The Ti_2p_ peak has a significant split spin-orbit component (Δ_metal_ = 6.1 eV). It is known that the splitting value can vary as a function of the chemical state (e.g., Δ_oxide_ = 5.7 eV). In the high-resolution XPS spectrum of Ti_2p_, we have two main peaks located at 459.34 eV (Ti_2p 3/2_) and at 465.14 eV (Ti_2p 1/2_) (Figure 5A). In this case, the split spin-orbit value is 5.8 eV, thus confirming the oxidation of the Ti film. The Ti_2p_ regions were symmetrical, indicating that Ti^4+^ was dominant on the surface [28]. Also, the Ti to O ratio of oxidized Ti films was 0.22 compared to the standard TiO_2_ stoichiometry, which presents a Ti/O ratio value of 0.5. This showed that many oxygen defects are present on the film surface. In addition, as we can see on the Si_2P_ high-resolution XPS spectrum, oxidized silicon may also contribute to O_1s_ signal intensity (Figure 5B). We can also conclude that the observed TiO_2_ is coming from partial oxidation of the Ti film deposited on SiNWs. Concerning the high-resolution spectrum of O_1s_, this can be fitted with two peaks. The peak at 530.90 eV corresponds to the lattice O (O–Ti–O), while the second peak at 533.14 eV was from the surface hydroxyl groups (Ti–OH) [29]. The peak corresponding to lattice O showed that Ti^4+^ was dominant in oxidized Ti film. In Figure 5D, we can see the C_1s_ contribution (8.7 atm. %), probably due to surface contamination by non-oxidized and oxidized carbon species with two peaks at 285.6 eV and 289.73 eV, respectively.

**Figure 6:**
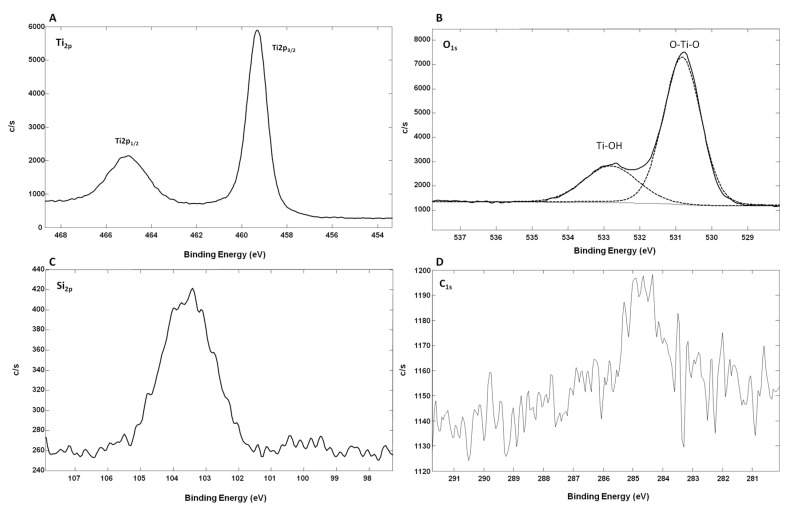
High-resolution XPS spectra of the TiO_2_-SiNWs’ (10 nm) surface, with Ti_2p_ (**A**), O_1s_ (**B**), Si_2p_ (**C**) and C_1s_ (**D**). (c/s = counts/second).

Figure 6 shows the high-resolution XPS spectra of TiO_2_-SiNWs. As for oxidized Ti-SiNWs, the high-resolution XPS spectrum of Ti_2p_ showed two main peaks located at 459.29 eV (Ti_2p 3/2_) and at 465.16 eV (Ti_2p 1/2_) (Figure 6A). Both the split spin-orbit value of 5.87 eV and the symmetrical Ti_2p_ regions showed that Ti^4+^ was also dominant on the surface, as already observed for oxidized Ti-SiNWs. However, the Ti to O ratio of TiO_2_ film was slightly higher, with a value of 0.31 compared to the standard TiO_2_ stoichiometry (Ti/O = 0.5), which correlates well with the deposition of TiO_2_ materials. Oxygen defects were still present, but with less influence of Ti–OH groups and SiO*x*, as we can see on Figure 6B,C. Concerning the high-resolution spectrum of O_1s_, this can also be fitted with two peaks (Figure 6B). The peak at 530.82 eV corresponds to the lattice O (O–Ti–O), while the second (less intense than for oxidized Ti-SiNWs) at 532.82 eV was from the surface hydroxyl groups (Ti–OH). The peak corresponding to lattice O showed that Ti^4+^ was dominant in oxidized Ti film. On Figure 6C, the Si_2P_ high-resolution XPS spectrum shows much lower signal intensity than for that obtained from oxidized Ti-SiNW (2.26 atm. % vs. 19.7 atm. %). This is probably due to the better surface coverage of SiNWs achieved by the ALD method. Finally, in Figure 6D we can also see the C_1s_ contribution, probably coming from surface contamination by non-oxidized and oxidized carbon species, with two peaks at 285.48 eV and 289.6 eV, respectively.

From EDX and XPS analyses, we have demonstrated that both deposition methods led to the coating of SiNWs by TiO_2_ layers. In the case of the TiO_2_-SiNW surface, we have demonstrated that ALD provides a high quality of conformal film deposition with lower amounts of hydroxyl groups present on the surface. The anatase phase of TiO_2_, which has been already characterized by XRD analysis in one of our recent studies should be noted [30]. Concerning the thermal evaporation of metallic titanium film, its exposure to ambient air at room temperature led to the spontaneous formation of a passive oxide film. It is known that this film is amorphous and composed of three layers (TiO, Ti_2_O_3_ and TiO_2_) with an overall thickness ranging from 5 nm to 10 nm. Ti_2_O_3_ is the intermediate layer, whereas the layer in contact with the environment is the anatase TiO_2_ layer [31].

**Figure 7:**
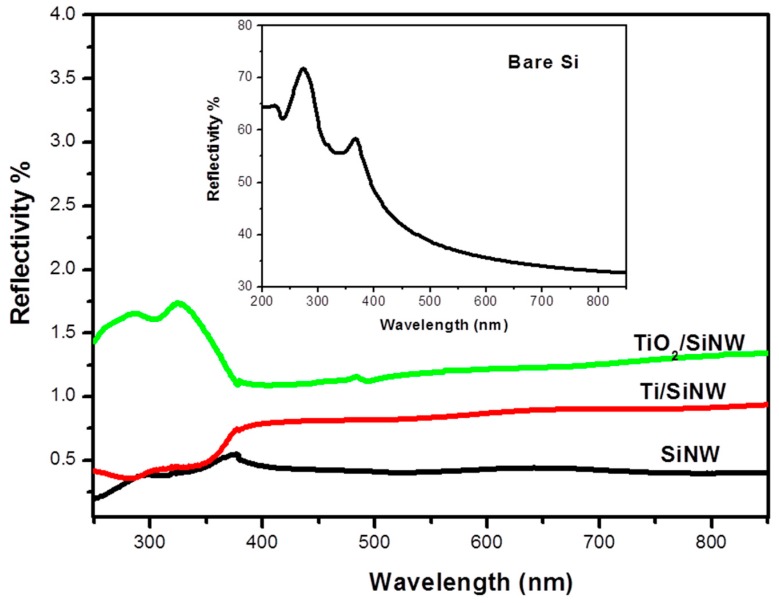
Reflectivity measurements of Si (insert), SiNWs, TiO_2_-SiNWs and oxidized Ti-SiNWs.

The optical properties of our interfaces were also assessed. To do so, we performed reflectivity measurements of oxidized Ti-SiNWs, TiO_2_-SiNWs and SiNWs surfaces. All surfaces exhibited anti-reflective properties at λ = 355 nm (mass spectrometer UV laser wavelength) compared to bulk silicon (Figure 7 and insert).

However, the SiNWs sample exhibited the lowest reflectance in the whole range (250–850 nm), with a value attaining 0.5% of reflectivity, as already shown in our previous work [22]. After Ti thermal evaporation and air oxidation, reflectivity was below 0.5% until 380 nm, and then increased slightly to 0.75% from 380 nm to 850 nm. By contrast, with TiO_2_ deposition by ALD (10 nm), a slight increase of the reflectivity was observed, with 1.5% of reflectivity in the spectral range 250–400 nm and 1.2% of reflectivity from 400 nm to 800 nm. Such anti-reflective surfaces will provide higher photon absorption during the LDI process.

MOAC is one of the most widely used techniques for phosphopeptide enrichment while limiting non-specific interactions. Titanium oxide (TiO_2_) has amphoteric properties, which means that it can behave as either a Lewis acid or a Lewis base as a function of the pH of its environment. Under acidic condition, TiO_2_ acts as a Lewis acid with positive charges that can display an ion-exchange feature [32]. The mechanism of interaction is believed to be based on both ion exchange and Lewis acid/base interactions, leading to high specificity [33]. However, as for the IMAC method, it is true that acidic residues can also bind TiO_2_ with high affinity, thereby capturing acidic peptides and phosphopeptides as well. Nevertheless, the addition of strong organic acids (like trifluoroacetic acid (TFA)) to the interaction buffer, minimizes the binding of carboxyl groups (from Aspartic acid or Glutamic acid for instance) to TiO_2_ and thus can prevent non-specific interactions resulting in greater specificity for phosphopeptides [17,34]. Indeed, at pH 2, acidic amino acid residues such as Aspartic acid (D) or Glutamic acid (E) will be protonated (E pKa ≈ 4.3 and D pKa ≈ 3.7) and no interactions could happen. By contrast, phosphate groups have two ionization steps. The first ionization is around pH ≈ 1.5 and the second around pH ≈ 6.3, meaning that at pH ≈ 2 phosphate groups have negative charge and can interact with the positively charged TiO_2_ surface. The ability of our interfaces to selectively capture phosphopeptides was then investigated. To do so, a phosphopeptide mixture composed of both non-phosphorylated and phophosrylated peptides was used in acetonitrile/water/TFA solution (50/49.9/0.1 *v*/*v*/*v*). Trifluoroacetic acid (TFA, 0.1% *v*/*v*, pH ≈ 2) was supplemented in the loading buffer to reduce non-specific interactions [17]. This mixture contained: Angiotensin II (1046.54 *m*/*z*, DRVYIHPF); Angiotensin I (1296.68 *m*/*z*, DRVYIHPFHL); Myelin basic protein, fragment 104–118 (1578.85 *m*/*z*, GKGRGLSLSRFSWGA); pTpY peptide (MAP kinase fragment 177–189) (1669.67 *m*/*z*, DHTGFL**pT**E**pY**VATR); pY peptide (insulin receptor, fragment 1142–1153) (1702.75 *m*/*z*, TRDI**pY**ETDYYRK); pT peptide (1720.89 *m*/*z*, VPIPGRFDRRV**pT**VE); pS peptide (PKA RII peptide, fragment 81–99) (2192.08 *m*/*z*, DLDVPIPGRFDRRV**pS**VAAE) (see Table 1).

**Figure 8:**
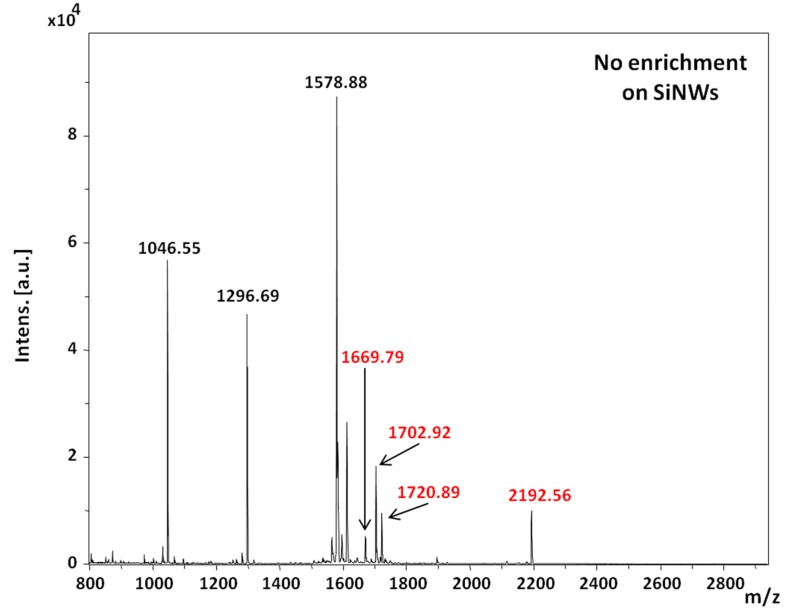
MS spectrum of phosphopeptides after deposition of 1 µL of the peptide mixture on SiNWs and allowed to dry.

The concentration varied from 400 fmol/µL to 4 fmol/µL. Then, different SiNWs with TiO_2_ or oxidized Ti films of different thicknesses were assessed. Thicknesses of 2 nm, 5 nm and 10 nm and 5 nm and 10 nm for TiO_2_ films deposited by ALD and oxidized Ti deposited by thermal evaporation followed by air oxidation, respectively, were used. When 1 µL was allowed to dry on all surfaces, no enrichment was obviously observed, as can be seen in Figure 8, where all the seven peptides were detected on the SiNWs surface.

Table 2 summarizes the enrichment results obtained from the phosphopeptide mixture (400 fmol/µL) on different surfaces. As can be seen, the best enrichment was obtained for the oxidized Ti-SiNWs’ surface with 10 nm of Ti, whereas the 10 nm TiO_2_-SiNWs’ surface was only able to detect three phosphopeptides. We can attempt to explain this by the fact that it is well known that the properties of TiO_2_ films (optical, thermal, electrical etc.) strongly depend on the conditions of the mode of film deposition, which leads to different surface behavior and performance [35,36]. In our case, the air oxidation of the metallic Ti layer induced an increased amount of hydroxyl groups compared to TiO_2_ deposited by ALD (Figure 5B and Figure 6B). However, at this stage, no assumption concerning the influence of hydroxyl groups on the phosphopeptide enrichment can be drawn. Other systematic studies (especially on the material itself) are needed to confirm this hypothesis.

From Table 2, we can also notice that the silicon nanowires covered by 2 nm of TiO_2_ (ALD) were not able to detect any phosphorylated peptide, whereas 5 nm of TiO_2_ allowed the detection of two phosphopeptides. This difference could be due to incomplete coverage of the SiNWs by TiO_2_. Indeed, even if the ALD deposition method allows a conformal coverage on well-defined structures such as on a periodic network of nanowires through lithography and dry etching, this is not the case in our study. In fact, since our surface morphology resembles strongly packed nanowires, we can suppose that, for low thicknesses, the overall nanowires are not totally covered by TiO_2_ film, thus explaining the lower performances. The same conclusion can be drawn when the enrichment performances for 5 nm and 10 nm of oxidized Ti films are compared.

**Figure 9:**
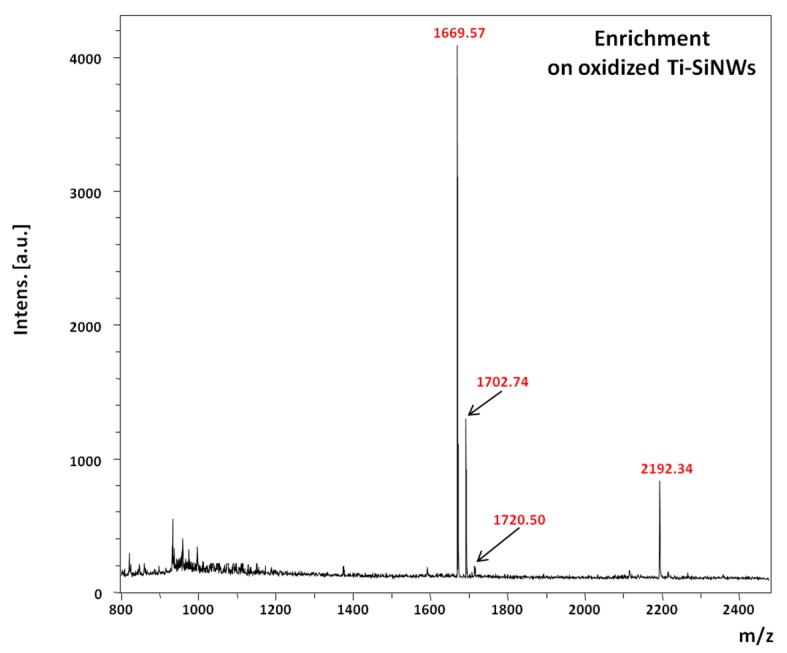
MS spectrum of phosphopeptide enrichment on oxidized Ti-SiNWs (10 nm).

Figure 9 shows the MS spectrum of phosphopeptide enrichment performed on oxidized Ti-SiNWs from 400 fmol/μL solution and after 10 min of incubation, rinsing and drying prior to LDI-MS. It should be noted that increasing the incubation time had no effect on the improvement of the detection sensitivity.

Despite the fact that the reproducibility and repeatability of SiNWs (without a Ti coating) was already proven, the same experiments have been performed on oxidized Ti-SiNWs as well [22,23]. All experiments were repeated at least three times to ensure satisfactory spectral reproducibilities (inter-spot, intra-spot and surface-to-surface variations). Good reproducibilities with a relative standard deviation (RSD) below 15% were obtained, except for the pT peptide (1720.86 *m*/*z*) for which RSD was much higher (RSD ≈ 40%). This is probably due to weaker interactions of the pT peptide with the interface because of its basic behavior (isolectric point ≈ 10.56). To assess the sensitivity of detection, a dilution of peptide mixture was performed to obtain a concentration of 40 and 4 fmol/µL. In both cases, oxidized Ti-SiNWs (10 nm) gave the best enrichment properties, i.e., the four phosphorylated peptides were detected. Nevertheless, some attempts were made at concentrations below 4 fmol/mL with modest success and poor reliability, meaning that below this concentration no satisfactory results can be reached.

**Figure 10:**
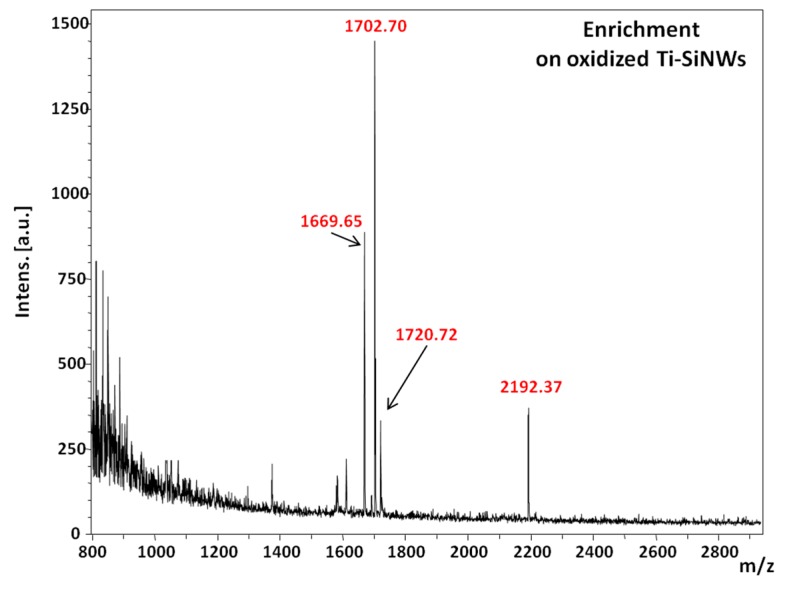
MS spectrum of phosphopeptide enrichment on oxidized Ti-SiNWs (10 nm) from serum.

Finally, the performance of the oxidized Ti-SiNWs (10 nm) interface was examined in a more complex biological sample. To do so, we used a solution of serum composed of 50% fetal bovine serum (FBS) in phosphate buffer saline (PBS) (*v*/*v*). Then, a spike of phosphopeptide mixture (to reach 400 fmol/µL), without any further treatment, was made. 3 µL of the resulting mixture was incubated on the oxidized Ti-SiNWs’ surface for 10 min, followed by appropriate rinsing and drying as already described above. Figure 10 shows the MS spectrum of the phosphopeptide mixture spiked in FBS solution. As we can see, only peaks related to [M + H]^+^ ions of phosphorylated peptides were detected at 1669.65 *m*/*z*, 1702.70 *m*/*z*, 1720.72 *m*/*z* and 2192.37 *m*/*z*.

## 3. Materials and Methods

### 3.1. Materials

Silicon wafers were purchased from Siltronix (Archamps, France). All cleaning and etching reagents were of VLSI grade. Sulphuric acid, 96% (H_2_SO_4_) was from Technic (Noyarey, France); hydrogen peroxide 30% (H_2_O_2_) and nitric acid 65% (HNO_3_) were from Carlo Erba (Milan, Italy); hydrofluoric acid 50% (HF) and ammonium fluoride (NH_4_F) were supplied by BASF (Ludwigshafen, Germany). All chemicals were of reagent grade or higher and were used as received unless otherwise specified. Silver nitrate (AgNO_3_), acetone, dichloromethane (CH_2_Cl_2_), isopropyl alcohol, acetonitrile (ACN), PBS tablets, and hexane were purchased from Sigma-Aldrich (St. Quentin Fallavier, France) and used without any further purification. Octadecyltrichlorosilane (OTS) was purchased from ABCR (Karlsruhe, Germany). Ultrapure water (Milli-Q, 18 MΩ·cm) was used for the preparation of the solutions and for all rinsing steps.

#### 3.1.1. Nanostructure Fabrication

Silicon nanowires were synthesized by metal-assisted chemical etching of *p*-type (100) crystalline silicon wafer (with a resistivity of 0.009–0.010 Ω·cm, Siltronix, Archamps, France) in NH_4_F/HNO_3_/AgNO_3_ aqueous solution [22,23]. The silicon surface was first degreased in acetone and isopropyl alcohol, rinsed with Milli-Q water, and then cleaned in a “piranha” solution (3/1 concentrated H_2_SO_4_/30% H_2_O_2_) for 20 min at 80 °C, followed by copious rinsing with Milli-Q water. The clean silicon surface was dipped in NH_4_F/HNO_3_/AgNO_3_ (6.00 M/5.73 M/0.02 M) aqueous solution at room temperature for 10 min. The resulting surface was rinsed copiously with deionized water and immersed in HNO_3_ (65%), with one rapid bath followed by a second bath of 10 min to ensure the removal of all silver particles and dendrites deposited on the surface during the chemical etching, and to reveal the silicon nanostructures. Finally, the surface was rinsed copiously with deionized water and dried under a gentle stream of nitrogen.

#### 3.1.2. Safety Considerations

The “piranha” solution is made of H_2_SO_4_/H_2_O_2_ with a 1:1 ratio (*v*/*v*). This is employed as a cleaning solution for silicon wafers prior to their use. As the “piranha” solution is a strong oxidant, it can react violently with organic materials, and can cause severe skin burns. It must be handled with extreme care in a well-ventilated fume hood with users wearing appropriate chemical safety protection.

Hydrofluoric acid (HF) is a hazardous acid that can result in serious tissue damage if burns are not appropriately treated. The etching of silicon should be performed in a well-ventilated fume hood with appropriate safety considerations: face shield and double-layered nitrile gloves.

#### 3.1.3. TiO_2_-SiNW Surface

TiO_2_ films were deposited using atomic layer deposition (ALD) on freshly prepared SiNWs. ALD allows the production of conformal coatings on substrates even on 1D objects such as SiNWs. TiO_2_ was deposited from TiCl_4_ as the titanium source and H_2_O vapor as the oxygen source (TFS200 Beneq, Espoo, Finland). Nitrogen gas was used as the purge gas and the chamber temperature and pressure were set to 260 °C and 2.8 mbars, respectively. TiCl_4_ and H_2_O pulse durations were 250 ms for both precursors separated by 2 s of N_2_ purge. The thicknesses of the TiO_2_ films were measured by ellipsometry on a control flat silicon surface.

The TiO_2_-SiNWs were then treated by UV/ozone (UV-O Cleaner, Jelight Company, Inc. (Irvine, CA, USA), 4 mW/cm^2^ at 220 nm) for 20 min to remove any organic contaminant from the surface and to generate surface hydroxyl groups. SiNWs were then immersed in a 10^−3^ M solution of OTS (octadecyltrichlorosilane) in hexane for 4 h at room temperature, in a dried nitrogen-purged glovebox. The resulting surfaces were rinsed with CH_2_Cl_2_, isopropyl alcohol, and dried under a gentle stream of nitrogen. TiO_2_-SiNWs display a superhydrophobic behavior (water contact angle (WCA) value > 150°). To ensure the access of phosphopeptides to the TiO_2_ surface, apertures were created. To do so, UV/O_3_ was performed for 60 min through an optical mask in patterns consisting of circles of 800 µm in diameter with an interspacing of 1800 µm. UV/O_3_ treatment removed the OTS layer and provided access for the TiO_2_. Then, the TiO_2_-SiNWs were composed of hydrophilic apertures with TiO_2_ accessible for interactions and surrounded by the OTS layer (superhydrophobic behavior) for liquid droplet confinement. All the steps are summarized in Figure 2.

#### 3.1.4. Ti-SiNWs’ Surface

##### Thermal Evaporation

Freshly prepared SiNWs were treated with UV/ozone (UV-O Cleaner, Jelight Company, Inc., 4 mW/cm^2^ at 220 nm) for 20 min to remove any organic contaminant from the surface and to generate surface hydroxyl groups. SiNWs were then immersed in a 10^−3^ M solution of OTS (ocatdecyltrichlorosilane) in hexane for 4 h at room temperature, in a dried nitrogen-purged glovebox. The resulting surfaces were rinsed with CH_2_Cl_2_, isopropyl alcohol, and dried under a gentle stream of nitrogen. SiNWs display superhydrophobic behavior (WCA value > 150°). To create patterns for making oxidized Ti spots, we used photolithography to remove the OTS locally. The steps are summarized in Figure 2. The same optical mask as for the TiO_2_-SiNWs was used, with patterns consisting of circles of 800 µm in diameter with an interspacing of 1800 µm. The patterns are created after exposure, post-bake and development. Oxygen plasma treatment was performed for 30 s to remove the resist remaining inside the apertures and to destroy the OTS layer locally. Then, Ti films were evaporated via thermal evaporation by electron beam base evaporation in Plassys MEB 550S (Bestek, Marolles-en-Hurepoix, France). Deposition was achieved using planetary rotation to ensure a good metallic coverage of the SiNWs. Finally, the lift-off was performed by dipping the surface in acetone at 35 °C for 20 min., rinsing with isopropanol, and drying under a nitrogen flow. The oxidized Ti-SiNWs were composed of hydrophilic apertures with oxidized Ti spots for interactions and surrounded by the OTS layer (superhydrophobic behavior) for liquid droplet confinement.

### 3.2. Characterization of Interfaces

#### 3.2.1. Scanning Electron Microscopy (SEM)

SEM images were obtained using an electron microscope ULTRA 55 (Zeiss, München, Germany) equipped with a thermal field emission emitter, three different detectors (EsB detector with filter grid; high efficiency In-lens SE detector; and Everhart-Thornley Secondary Electron Detector) and an EDX device.

#### 3.2.2. Reflectivity Measurement

The reflectance measurements were performed using a UV-Vis spectrophotometer (Perkin-Elmer Lambda UV-Vis 950 spectrophotometer, Villebon-sur-Yvette, France) equipped with an integrating sphere. The scans were measured for wavelength ranging from 250 nm to 850 nm at an incident light angle of 45° and at different locations on each scanned surface.

#### 3.2.3. Transmission Electron Microscopy (TEM)

First of all, a slice of 100 nm thick of TiO_2_-SiNW was prepared using a focused ion beam (FIB). To do so, hydrogen silesquoxane (HSQ) resist was spin-coated on the TiO_2_-SiNW surface followed by rapid annealing at 80 °C to embed all the nanostructures. Then, the slice was achieved through FIB and deposited on a 3 mm diameter copper grid. Transmission electron microscopy and selected area electron diffraction patterns (SAED) were recorded on a CM 30 Philips operating at 300 kV and equipped with an EDX detector.

#### 3.2.4. X-ray Photoelectron Spectroscopy (XPS)

For XPS measurements, we used a monochromatic Al Kα X-ray source and an analyzer pass energy of 12 eV. Under these conditions, the overall resolution as measured from the full width at half-maximum (fwhm) of the Ag_3d 5/2_ line was 0.55 eV. The binding energy scale was calibrated using the Au_4f 7/2_ line at 84.0 eV (Calibration). The acceptance angle of the analyzer was set to 14°, and the angle between the incident X-rays and the analyzer was 90°. The detection angle of the photoelectrons was 25°, as referenced to the sample surface. The intensities of the various XPS core levels (CLs) were measured as the peak area after standard background subtraction according to the Shirley procedure. For the CL decomposition, we used Voigt functions and a least squares minimization procedure. The different components were modeled with the same parameters i.e., the Gaussian and Lorentzian broadenings were kept fixed for each component of a given CL.

#### 3.2.5. Contact Angle Measurements

Water contact angles were measured using deionized water. We used a remote-computer controlled goniometer system (DIGIDROP by GBX, Romans sur Isère, France) for measuring the contact angles. The accuracy was ±2°. All measurements were made in ambient atmosphere at room temperature.

#### 3.2.6. Sample Preparation

The phosphopeptides were purchased from Molecular Probes (Invitrogen, Paris, France). Then, different solutions of phosphopeptides were prepared in ACN/H_2_O/TFA (50/49.9/0.1, *v*/*v*/*v*) (incubation buffer) and diluted as needed with same buffer. Spikes of phosphopeptides were achieved by adding a certain volume of phosphopeptide stock solution to diluted FBS from Gibco in phosphate buffer saline (PBS) @50% (*v*/*v*).

#### 3.2.7. Specific Capture

For the specific capture of phosphopeptides on oxidized Ti/TiO_2_-SiNWs, a droplet of the sample (3 µL) was deposited and allowed to incubate for 30 min in a home-made incubation chamber with a water-saturated atmosphere at room temperature to prevent droplet evaporation. After incubation, the droplet of sample was removed by capillarity (absorbed by a piece of tissue), followed by several rinsing steps with 3 × 200 µL of incubation buffer (ACN/H_2_O/TFA 50/49.9/0.1 *v*/*v/v*) and by 2 × 200 µL of deionized water by using a micropipette. It is important to prevent the surface from drying during the rinsing steps. Finally, 1 µL of ammonium citrate (1 mM) was added on each spot and was allowed to dry prior to MS detection.

#### 3.2.8. LDI-MS Analysis

MALDI mass spectra were recorded on an Ultraflex TOF/TOF instrument (Bruker Daltonics, Wissembourg, France) equipped with LIFT capability. A pulsed Nd:YAG laser at a wavelength of 355 nm (98% focus) was operated at a frequency of 100 Hz with a delayed extraction time of 30 ns. The source was operated in the positive mode. An acceleration voltage of 25.0 kV (IS1) was applied for a final acceleration of 21.95 kV (IS2). The reflectron mode was used for the TOF analyzer (voltages of 26.3 kV and 13.8 kV). Random laser irradiation was performed according to the partial sample mode with 10 shots/point. One acquisition was recorded from 5000 shots corresponding to 500 irradiated positions, the laser fluence being adjusted for each studied sample. Ions were detected over a mass range from 800–3000 *m*/*z*. We chose to fix the S/N threshold at 3 for more reliability. Data were acquired with Flex Control software and processed with Flex Analysis software. The SiNWs’ interfaces were fixed at the corners with a conductive tape on an MTP TLC adapter (Bruker Daltonics, Wissembourg, France). The plate was then introduced into the mass spectrometer ion source. External calibration was performed using a commercial peptide mixture (calibration peptide standard 1 or 2, Bruker Daltonics, Wissembourg, France).

## 4. Conclusions

In this work we have demonstrated, for the first time, the coating of silicon nanowires by TiO_2_-based films for “on plate” phosphopeptide enrichment and their detection by mass spectrometry. One type of coating was achieved by the thermal evaporation of Ti metallic film followed by its air oxidation, while a second was achieved by atomic layer deposition. Different thicknesses were also deposited. After their characterization by EDX, XPS and reflectivity measurements, all TiO_2_ coatings presented Ti^4+^ ions and anti-reflective behavior. We then assessed their ability, specifically, to enrich phosphopeptides from a peptide mixture for subsequent detection by matrix-free laser desorption/ionization mass spectrometry. The best results were obtained from 10 nm-thick oxidized Ti film (thermal evaporation), which was able to enrich all phosphopeptides and allowed their LDI-MS detection. This “one-plate” enrichment was also done for spiked serum, proving that our surface can be used for specific capture in a complex medium followed by MS detection without any further sample preparation.

## Figures and Tables

**Table 1:** Peptide mixture used in this study. pX (in red) corresponds to phosphorylated amino acid with pS: phosphorylated Serine; pT: phosphorylated Threonine; and pY: phosphorylated Tyrosine.

Peptides	M + 1	Sequence
Angiotensin II	1046.54	DRVYIHPF
Angiotensin I	1296.68	DRVYIHPFHL
Myelin basic protein, fragment 104–118	1578.85	GKGRGLSLSRFSWGA
pTpY peptide (MAP kinase fragment 177–189)	1669.67	DHTGFLpTEpYVATR
pY peptide (insulin receptor, fragment 1142–1153)	1702.75	TRDIpYETDYYRK
pT peptide	1720.89	VPIPGRFDRRVpTVE
pS peptide (PKA RII peptide, fragment 81–99)	2192.08	DLDVPIPGRFDRRVpSVAAE

**Table 2:** Phosphopeptide enrichment and MS detection achieved on different surfaces investigated in this work.

Layers on SiNWs	Thickness (nm)	Deposition Technique	Enrichment	Number of Phosphopeptides Detected
Oxidized Ti	5	Evaporation	Yes	3
Oxidized Ti	10	Evaporation	Yes	4
TiO_2_	2	ALD	No	0
TiO_2_	5	ALD	Yes	2
TiO_2_	10	ALD	Yes	3
SiNWs	-	-	No	All peptides detected

## References

[1] Morandell S., Stasyk T., Grosstessner-Hain K., Roitinger E., Mechtler K., Bonn G.K., Huber L.A. (2006). Phosphoproteomics strategies for the functional analysis of signal transduction. Proteomics.

[2] Reinders J., Sickmann A. (2005). State-of-the-art in phosphoproteomics. Proteomics.

[3] Graves J.D., Krebs E.G. (1999). Protein phosphorylation and signal transduction. Pharmacol. Ther..

[4] Hunter T. (2000). Signaling–2000 and beyond. Cell.

[5] Thingholm T.E., Jensen O.N., Larsen M.R. (2009). Analytical strategies for phosphoproteomics. Proteomics.

[6] Rush J., Moritz A., Lee K.A., Guo A., Goss V.L., Spek E.J., Zhang H., Zha X.M., Polakiewicz R.D., Comb M.J. (2005). Immunoaffinity profiling of tyrosine phosphorylation in cancer cells. Nat. Biotechnol..

[7] Hinsby A.M., Olsen J.V., Mann M. (2004). Tyrosine phosphoproteomics of fibroblast growth factor signaling: A role for insulin receptor substrate-4. J. Biol. Chem..

[8] Tao W.A., Wollscheid B., O’Brien R., Eng J.K., Li X.J., Bodenmiller B., Watts J.D., Hood L., Aebersold R. (2005). Quantitative phosphoproteome analysis using a dendrimer conjugation chemistry and tandem mass spectrometry. Nat. Methods.

[9] Li X., Gerber S.A., Rudner A.D., Beausoleil S.A., Haas W., Villen J., Elias J.E., Gygi S.P. (2007). Large-scale phosphorylation analysis of α-factor-arrested *Saccharomyces cerevisiae*. J. Proteome Res..

[10] Blacken G.R., Volny M., Vaisar T., Sadilek M., Turecek F. (2007). In Situ enrichment of phosphopeptides on MALDI plates functionalized by reactive landing of zirconium(IV)-*n*-propoxide ions. Anal. Chem..

[11] Larsen M.R., Thingholm T.E., Jensen O.N., Roepstorff P., Jorgensen T.J. (2005). Highly selective enrichment of phosphorylated peptides from peptide mixtures using titanium dioxide microcolumns. Mol. Cell. Proteom..

[12] Imanishi S.Y., Kochin V., Ferraris S.E., de Thonel A., Pallari H.M., Corthals G.L., Eriksson J.E. (2007). Fast and reliable phosphopeptide validation by microLC-ESI-Q-TOF MS/MS. Mol. Cell. Proteom..

[13] Li Y., Leng T., Lin H., Deng C., Xu X., Yao N., Yang P., Zhang X. (2007). Preparation of Fe_3_O_4_@ZrO_2_ core-shell microspheres as affinity probes for selective enrichment and direct determination of phosphopeptides using matrix-assisted laser desorption ionization mass spectrometry. J. Proteome Res..

[14] Zhou H., Ye M., Dong J., Han G., Jiang X., Wu R., Zou H. (2008). Specific phosphopeptide enrichment with immobilized titanium ion affinity chromatography adsorbent for phosphoproteome analysis. J. Proteome Res..

[15] Loyet K.M., Stults J.T., Arnott D. (2005). Mass spectrometric contributions to the practice of phosphorylation site mapping through 2003: A literature review. Mol. Cell. Proteom..

[16] Dunn J.D., Igrisan E.A., Palumbo A.M., Reid G.E., Bruening M.L. (2008). Phosphopeptide enrichment using MALDI plates modified with high-capacity polymer brushes. Anal. Chem..

[17] Eriksson A., Bergquist J., Edwards K., Hagfeldt A., Malmström D., Hernandez V.A. (2010). Optimized Protocol for On-Target Phosphopeptide Enrichment Prior to Matrix-Assisted Laser Desorption-Ionization Mass Spectrometry Using Mesoporous Titanium Dioxide. Anal. Chem..

[18] Law K.P., Larkin J.R. (2011). Recent advances in SALDI-MS techniques and their chemical and bioanalytical applications. Anal. Bioanal. Chem..

[19] Wei J., Buriak J.M., Siuzdak G. (1999). Desorption-ionization mass spectrometry on porous silicon. Nature.

[20] Coffinier Y., Kurylo I., Drobecq H., Szunerits S., Melnyk O., Zaitsev V.N., Boukherroub R. (2014). Decoration of silicon nanostructures with copper particles for simultaneous selective capture and mass spectrometry detection of His-tagged model peptide. Analyst.

[21] Bi H., Qiao L., Busnel J.M., Devaud V., Liu B., Girault H.H. (2009). TiO_2_ Printed Aluminum Foil: Single-Use Film for a Laser Desorption/Ionization Target Plate. Anal. Chem..

[22] Dupré M., Coffinier Y., Boukherroub R., Cantel S., Martinez J., Enjalbal C. (2012). Laser desorption ionization mass spectrometry of protein tryptic digests on nanostructured silicon plates. J. Proteom..

[23] Dupré M., Enjalbal C., Cantel S., Martinez J., Megouda N., Hadjersi T., Boukherroub R., Coffinier Y. (2012). Investigation of silicon-based nanostructure morphology and chemical termination on laser desorption ionization mass spectrometry performance. Anal. Chem..

[24] Piret G., Drobecq H., Coffinier Y., Melnyk O., Boukherroub R. (2010). Matrix-free laser desorption/ionization mass spectrometry on silicon nanowire arrays prepared by chemical etching of crystalline silicon. Langmuir.

[25] Nguyen T.P.N., Coffinier Y., Thomy V., Boukherroub R. (2013). Fabrication of silicon nanostructures using metal-assisted etching in NaBF_4_. Phys. Status Solidi A.

[26] Kurylo I., Dupré M., Cantel S., Enjalbal C., Drobecq H., Szunerits S., Melnyk O., Boukherroub R., Coffinier Y. (2017). Characterization of peptide attachment on silicon nanowires by X-ray photoelectron spectroscopy and mass spectrometry. Analyst.

[27] Peterson S.A. (2007). Matrix-free methods for laser desorption/ionization mass spectrometry. Mass Spectrom. Rev..

[28] Hashimoto S., Tanaka A. (2002). Alteration of Ti_2p_ XPS spectrum for titanium oxide by low-energy Ar ion bombardment. Surf. Interface Anal..

[29] Liu B., Wen Q.H.L., Zhao X. (2009). The effect of sputtering power on the structure and photocatalytic activity of TiO_2_ films prepared by magnetron sputtering. Thin Solid Films.

[30] Hamdi A., Boussekey L., Roussel P., Addad A., Boukherroub R., Ezzaouia H., Coffinier Y. (2016). Hydrothermal preparation of MoS_2_/TiO_2_/Si nanowires composite with enhanced photocatalytic performance under visible light. Mater. Des..

[31] Feng B., Weng J., Yang B.C., Chen J.Y., Zhao J.Z., He L., Qi S.K., Zhang X.D. (2003). Surface characterization of titanium and adsorption of bovine serum albumin. Mater. Charact..

[32] Nawrocki J., Rigney J., McCormick A., Carr P.W. (1993). Chemistry of zirconia and its use in chromatography. J. Chromatogr. A.

[33] Leitner A. (2016). Enrichment Strategies in Phosphoproteomics. Methods Mol. Biol..

[34] Gates M.B., Tomer K.B., Deterding L.J. (2010). Comparison of metal and metal oxide media for phosphopeptide enrichment prior to mass spectrometric analyses. J. Am. Soc. Mass Spectrom..

[35] Svitasheva S.N., Gritsenko V.A., Kolesov B.A. (2008). Optical properties of TiO_2_ films made by air oxidation of Ti. Phys. Stat. Sol. C.

[36] Llansola-Portoles M.L., Bergkamp J.J., Finkelstein-Shapiro D., Sherman B.D., Kodis G., Dimitrijevic N.M., Gust D., Moore T.A., Moore A.L. (2014). Controlling Surface Defects and Photophysics in TiO_2_ Nanoparticles. J. Phys. Chem. A.

